# Investigating the association between the COVID-19 vaccination and incident gastrointestinal symptomology: A comprehensive dataset

**DOI:** 10.1016/j.dib.2023.109287

**Published:** 2023-06-01

**Authors:** Kaly D. Houston, Josette Hartnett, Suzanne J. Rose

**Affiliations:** Department of Research and Discovery, Stamford Hospital, Stamford, CT, USA

**Keywords:** COVID-19, Vaccine, Gastrointestinal Disorders

## Abstract

The coronavirus disease of 2019 (COVID-19) pandemic created a variety of symptoms from mild to acute in the general population. Additional disease burden was experienced in high-risk populations, such as older adults, people with disabilities or overweight, those from racial and ethnic minority groups, and patients with cancer, chronic kidney, lung or liver disease, or diabetes. Although it is well-known that SARS-CoV-2 mostly affects the respiratory tract, studies have revealed the presence of gastrointestinal (GI) symptoms in those patients diagnosed with COVID-19. The best protection against infection is through receipt of the COVID-19 vaccine, which is associated with a low incidence of adverse events. However, there is limited research on the lesser-known side effects experienced following receipt of the COVID-19 vaccination, amongst healthy and special needs populations.

This study investigated the association between the COVID-19 vaccination and, when it occurred, infection, and resulting gastrointestinal (GI) symptomology, focusing on both the general population and on those previously diagnosed with GI disorders, Irritable Bowel Syndrome (IBS) and Inflammatory Bowel Disease (IBD). Through a short, anonymous survey, 215 participants were assessed for acute onset of GI issues and/or worsening of pre-existing GI issues following the receipt of one or more COVID-19 vaccine doses and following contraction of COVID-19 itself, when applicable. All analyses were performed using SAS version 9.4, and prior to study initiation, the study protocol was reviewed and approved as exempt by the Stamford Hospital's Institutional Review Board of record.

Data analysis included reporting of demographic variables as well as descriptive statistics regarding side effects experienced after receipt of the COVID-19 vaccine, as well as after contracting COVID-19, if it occurred. To assess for statistically significant differences between the groups, ANOVA was conducted for each survey item. Reporting of results consisted of the mean and standard deviation within each of the groups, and an omnibus *p*-value less than 0.05 (*p* <0.05) was considered statistically significant. For the purposes of this report, a greater than 0.50 response difference between highest and lowest mean value will be presented. In the event of a statistically significant omnibus *p*-value, the Scheffe test was used as the post-hoc procedure.

The database created through this research demonstrates the prevalence of post-COVID-19 vaccination side effects and can serve as preliminary data for gaining a better understanding of how both general and populations with a higher disease burden are being affected by the COVID-19 vaccine, booster doses, and incident COVID-19 infection in vaccinated individuals.

**Specifications Table**

**Table:** 

Subject	Health and medical sciences
Specific subject area	This data assesses changes in gastrointestinal (GI) symptomology following receipt of COVID-19 vaccination(s) and when applicable, contraction of COVID-19. Data includes survey responses from both the general population and high-risk populations, here defined as those with previously diagnosed functional GI disorders (IBD and/or IBS).
Type of data	Table
How data were acquired	After Institutional Review Board (IRB) review, approval, and exempt determination, a comprehensive questionnaire to assess changes in GI symptomology prior to and following receipt of COVID-19 vaccination(s) and when applicable, contraction of COVID-19, was designed. Response items were multiple choice, as well as Likert-scale in nature. De-identified data was collected via SurveyMonkey into a secure database, and subsequently analyzed.
Data format	Microsoft Excel raw database housed in repository: https://doi.org/10.3886/E183721V4.
Description of data collection	Convenience sampling was used to collect de-identified survey data via a comprehensive, multiple-choice questionnaire distributed to all staff at Stamford Hospital. The survey response data was collected into a secure database and analyzed.
Data source location	Institution: Stamford Health City/Town/Region: Stamford, Connecticut Country: United States (US) Latitude and longitude (and GPS coordinates, if possible) for collected samples/data: 41° 3′ 10″ N, 73° 32′ 20″ W
Data accessibility	Repository name: Inter-university Consortium for Political and Social Research (ICPSR), openICPSR. Direct URL to data: https://www.openicpsr.org/openicpsr/project/183721/version/V4/view. Citation: Houston, Kaly D, Hartnett, Josette, and Rose, Suzanne J. Investigating the Prevalence of Gastrointestinal Symptomology Following Receipt of the COVID-19 Vaccine. Ann Arbor, MI: Inter-university Consortium for Political and Social Research, 2023-01-27. https://doi.org/10.3886/E183721V4 [1]. Data for this research are accessible upon registering for a free account via: https://www.icpsr.umich.edu/rpxlogin.

## Value of the Data

• The COVID-19 outbreak was declared a global pandemic in March of 2020 by the World Health Organization (WHO) [2] and presented novel challenges for the general and high-risk populations in regard to management and treatment of symptoms and illness [3,4]. While the virus causing COVID-19 mainly infects the respiratory tract, it has also been shown to cause gastrointestinal (GI) symptoms as well [5], [6], [7], [8]. Although the COVID-19 vaccination has been deemed safe [9,10] and able to alleviate the degree of new and acute infections, research on the side effects experienced, specifically GI symptomology, following the receipt of the COVID-19 vaccination is limited particularly for people with pre-existing medical conditions [8,[11], [12], [13], [14], [15].
• The structural decision to incorporate the analysis of GI symptomology following the receipt of each dose of the COVID-19 vaccination series, as well as symptomology following contraction of COVID-19, provides robust data that aids in understanding how populations are being affected.
• This data can be used as preliminary information to guide supplementary vaccine research related to GI disorders or symptomology, as well as used in preparation for facing future surges of COVID-19 and/or in the case of other pandemics of comparable nature. The insight provided here on less common side effects of COVID-19 vaccination can craft future treatment modalities towards mitigating the side effects of vaccines in both healthy and special needs patient populations.

## 1 Objective

There is limited research on the side effects experienced following receipt of the COVID-19 vaccination, especially among special needs populations. This study investigates an association between changes in symptomology, specifically gastrointestinal symptomology, following both contraction of COVID-19 and/or receipt of a single or multiple doses of the COVID-19 vaccination series. The purpose of this study is to gain a better understanding of how both the general and high-risk populations are being affected to aid in the preparation for future surges of COVID-19 and/or in the case of other pandemics with similar catastrophic effects to multiple organ systems.

## 2 Data Description

A total of 215 respondents were included in the analysis. Baseline demographics including age, gender, ethnicity, education, BMI and historical IBD/IBS diagnosis can be found in Table 1. Most participants were between 31 and 65 years, female (86%), and Caucasian (74.63%). Participants ranged in education level, with some (34.44%) having a bachelor's degree vs 25% having a master's degree. Almost 21% of the sample were either moderately obese or obese, and 173 respondents (86.07%) stated not having a diagnosis of IBS/IBD as compared to 28 who did (13.93%) (*p* <0.0001).

**Table 1:** Demographics of survey respondents.

Variable	Category	Count (%)	*p*-value
Age	16–30 years	33 (16.42%)	<0.0001
	31–45 years	77 (38.31%)	
	45–65 years	72 (35.82%)	
	65+ years	19 (9.45%)	

Gender	Female	173 (86.07%)	<0.0001
	Male	26 (12.94%)	

Ethnicity	African American	10 (4.98%)	<0.0001
	Asian	13 (6.47%)	
	Caucasian	150 (74.63%)	
	Hispanic or Latino	18 (8.96%)	

Education	High School	35 (17.41%)	<0.0001
	Bachelor's Degree	69 (34.33%)	
	Master's Degree	51 (25.37%)	
	PhD or higher	27 (13.43%)	

Body Mass Index	18.5- 24.9 (ideal weight)	95 (47.26%)	<0.0001
	25–29.9 (overweight)	66 (32.82%)	
	30–34.9 (moderate obesity)	21 (10.45%)	
	35–40+ (obesity)	19 (9.45%)	

IBS and/or IBD Diagnosis	Diagnosed	28 (13.93%)	<0.0001
	Not Diagnosed	173 (86.07%)	

Table 2 presents the results of GI symptomology following COVID-19 vaccination. 133 respondents (73.89%) reported experiencing side effects of which, 44 of them (33.85%) experienced a change in GI activity (*p* = 0.0002). 48.16% of participants attributed changes in GI activity to the second dose and 35.53% to their third dose (*p*<0.0001). Significant differences were not found in responses to vaccine doses among frequent bowel movements (*p* = 0.08), looser stool (or more diarrhea) (*p* = 0.47), more gas (*p* = 0.68), nor more pain while passing stool (*p* = 0.86). However, a significant difference was seen with experiencing a mix of diarrhea and constipation from two doses of the vaccine (*n* = 9, 29.03%) as compared to single dose (*n* = 6, 19.35%) (*p* = 0.02). Similarly, 7 respondents (25%) mentioned the presence of mucus in stool compared to 21 respondents (75%) (*p* = 0.0008) and 7 respondents reported the presence of blood in stool compared to 23 respondents (76.67%) (*p* = 0.003). There was a significant difference in response to the onset of changes in GI activity post-vaccination with 25% (*n* = 28) indicating changes within 1–2 days post-vaccination, followed by 13.39% (*n* = 15) within 3–7 days and lastly, 6.25% (*n* = 7) within 1–2 weeks (*p*=<0.0001).

**Table 2:** Gastrointestinal symptoms post-COVID-19 vaccination.

Variable	Category	Count (%)	*p*-value
Did you experience any side effects following any doses of the COVID-19 vaccine?	Yes	133 (73.89%)	<0.0001
	No	47 (26.11%)	

Do you recall experiencing a change in gastrointestinal activity following any doses of the COVID-19 vaccine?	Yes	44 (33.85%)	0.0002
	No	86 (66.15%)	

Indicate which doses resulted in changes in gastrointestinal activity	First Dose	19 (25%)	<0.0001
	Second Dose	29 (48.16%)	
	Third Dose	27(35.53%)	
	Fourth Dose	1 (1.31%)	

More frequent bowel movements	First Dose	8 (25%)	0.08
	Second Dose	5 (15.63%)	
	Third Dose	5 (15.63%)	

Looser stool (or more diarrhea)	First Dose	7 (21.21%)	0.47
	Second Dose	8(24.24%)	
	Third Dose	6 (18.18%)	

Mix of diarrhea and constipation	First Dose	6 (19.35%)	0.02
	Second Dose	9 (29.03%)	
	Third Dose	2 (6.45%)	

More gas	First Dose	9 (28.13%)	0.68
	Second Dose	9 (28.13%)	
	Third Dose	5 (15.63%)	

Presence of mucus in stool	Yes	7 (25%)	0.008
	N/A	21 (75%)	

Presence of blood in stool	Yes	7 (23.33%)	0.003
	N/A	23 (76.67%)	

More pain while passing stool	Yes	15 (48.39%)	0.86
	N/A	15 (48.39%)	

When did these changes in gastrointestinal activity start to occur following vaccination?	1–2 days	28 (25%)	<0.0001
	3–7 days	15 (13.39%)	
	1–2 weeks	7 (6.25%)	
	2–4 weeks	10 (8.93%)	
	More than a month	13 (11.61%)	

Responses regarding COVID-19 infection symptomology post-vaccination can be found in Table 3. 68 respondents (41.72%) contracted an acute COVID-19 infection post-vaccination as compared to 95 respondents (58.28%) (*p* = 0.03). Out of those 68 respondents, 12 (17.65%) reported changes in bowel movements both during and after contraction of infection, compared to 43 (63.24%) with no changes in bowel movements (*p*<0.0001). The most prominent nature of the change in GI activity following contraction of COVID-19 was more frequent bowel movements (27.58%), with the least prominent being both the presence of mucus in stool (7.41%) and more pain while passing stool (7.41%) (*p*<0.0001). 9 participants (36%) reported changes in GI activity persisting for 2–7 days as compared to 1 participant for 2–4 weeks (*p* = 0.04).

**Table 3:** COVID-19 infection symptomology post-vaccination.

Variable	Category	Count (%)	*p*-value
Have you contracted COVID-19?	Yes	68 (41.72%)	0.03
	No	95 (58.28%)	

Do you recall experiencing a change in bowel movement habits during or following COVID-19 infection(s)?	Yes, during acute infection	10 (14.71%)	<0.0001
	Yes, after acute infection	3 (4.41%)	
	Yes, both during and after infection	12 (17.65%)	
	No	43 (63.24%)	

The nature of the change in gastrointestinal activity following contraction of COVID-19	More frequent bowel movements	15 (27.58%)	<0.0001
	Looser stool (or more diarrhea)	14 (25.93%)	
	Harder stool	0	
	Mix of diarrhea and constipation	7 (12.96%)	
	More gas	10 (18.52%)	
	Presence of mucus in stool	4 (7.41%)	
	Presence of blood in stool	0	
	More pain while passing stool	4 (7.41%)	

How long did the changes in GI activity last?	1 or 2 days	2 (8%)	0.04
	2–7 days	9 (36%)	
	1–2 weeks	5 (20%)	
	2–4 weeks	1 (4%)	
	More than 3 months	8 (32%)	

Table 4 presents the results of symptoms post-COVID-19 vaccination with a focus on side effects specifically pertaining to stomach pain and nausea. 30 respondents (25.21%) experienced new stomach pain or changes in frequency of stomach pain compared to 89 respondents (74.79%) who did not (*p*<0.0001). 19 respondents (39.58%) indicated that the change in frequency of stomach pain followed the third dose of the vaccine while 3 respondents (6.25%) attributed changes to the fourth dose (<0.0001). Following the receipt of COVID-19 vaccination, the onset of stomach pain differed significantly among participants, with 43.14% reporting onset within 1–2 days post-vaccination compared to 5.88% within 1–2 weeks (*p* = 0.04). 23 participants (47.92%) indicated that their stomach pain persisted for more than 3 months, while 2 participants (4.17%) reported 2–4 weeks (*p* = 0.01). Similarly, 17 respondents (14.66%) experienced nausea post-vaccination as compared to 99 respondents (85.34%) (*p*<0.0001). There were no significant differences between specific doses of the vaccine and resulting changes in frequency of nausea (*p* = 0.21), as well as for the onset of nausea following vaccination (*p* = 0.33). However, there was a significant difference in duration of nausea symptomology with 12 participants (36.35%) reporting persistence for 1–2 days, followed by 9 participants (27.27%) for more than three months and 2 participants (6.06%) for 1–3 months (*p* = 0.02).

**Table 4:** COVID-19 infection symptomology post-vaccination:stomach pain and nausea.

Variable	Category	Count (%)	*p*-value
Do you recall experiencing new stomach pain or a change in frequency of stomach pain?	Yes	30 (25.21%)	<0.0001
	No	89 (74.79%)	

Changes in frequency of stomach pain	First Dose	8 (16.67%)	<0.0001
	Second Dose	18 (37.5%)	
	Third Dose	19 (39.58%)	
	Fourth Dose	3 (6.25%)	

When did this change in frequency of stomach pain start to occur, following vaccination?	1–2 days	22 (43.14%)	0.04
	3–7 days	16 (31.37%)	
	1–2 weeks	3 (5.88%)	
	2–4 weeks	4 (7.84%)	
	More than a month	6 (11.76%)	

Duration of changes in frequency of stomach pain	1–2 days	8 (16.67%)	0.01
	3–7 days	7 (14.58%)	
	2–4 weeks	2 (4.17%)	
	1–3 months	8 (16.67%)	
	More than 3 months	23 (47.92%)	

Do you recall experiencing a change in frequency of nausea following any doses of the COVID-19 vaccine?	Yes	17 (14.66%)	<0.0001
	No	99 (85.34%)	

Indicate which of the following doses resulted in changes in frequency of nausea.	First Dose	10 (30.3%)	0.21*
	Second Dose	15 (45.45%)	
	Third Dose	8 (24.24%)	
	Fourth Dose	0	

When did this change in frequency of nausea start to occur, following vaccination?	1–2 days	20 (58.82%)	0.33
	3–7 days	5 (14.71%)	
	1–2 weeks	1 (2.94%)	
	More than a month	8 (23.53%)	
How long did these changes in frequency of nausea last?	1–2 days	12 (36.36%)	0.02*
	3-7 days	6 (18.18%)	
	1-2 weeks	4 (12.12%)	
	1-3 months	2 (6.06%)	
	More than 3 months	9 (27.27%)	

## 3 Experimental Design, Material and Methods

After Institutional Review Board review and exempt determination, the authors utilized a cross-sectional survey design to assess the presence and nature of side effects following both the receipt of the COVID-19 vaccination and side effects after contracting COVID-19. A comprehensive, 40-item questionnaire was administered among a convenience sample of healthcare workers at a community-based teaching hospital. The questionnaire incorporated multiple-choice response options with “skip logic”, allowing for an adaptive quality to guide respondents to the appropriate questions based on their previous responses.

The first portion of the survey prompted respondents to report their vaccination status to determine if they met the inclusion criteria of having received at least one dose of the COVID-19 vaccination series. Those deemed eligible were then directed to report the following demographic information: age group, Body Mass Index, gender identity, marital status, ethnicity, and highest degree/level of education completed. Respondents who identify their ethnicity as White were prompted to select “Caucasian”. Pertaining to their medical history, respondents were asked to report whether they had ever been diagnosed with IBD or IBS. If so, the year of diagnosis was collected along with the report of any “flare” of their pre-existing IBD or IBS symptoms following any doses of the COVID-19 vaccination series and/or a COVID-19 infection. The current study findings are representative of the general population diagnosed with IBS or IBD as it is estimated that 10–15% and 1.3% of the US population has been diagnosed with IBS and IBD, respectively [16,17].

From here, both the date of receipt and manufacturer of each given dose of the COVID-19 vaccination series was requested. Using “skip logic”, the questionnaire allowed respondents to report up to four doses and guided them to the next section if reported that they did not receive a given dose.

Next, the questionnaire inquired about the presence of general side effects following the receipt of the COVID-19 vaccine. A dropdown menu allowed respondents to indicate which dose(s), if any, elicited any of the following side effects: reaction at the injection arm (e.g. pain, redness, swelling), tiredness throughout the body, headache, muscle pain throughout the body, chills, and fever. The duration of the side effects post-vaccination was also inquired in the following increments: 1–2 days, 3–7 days, more than a week, or not applicable (N/A) if not experienced.

In a similar fashion, the presence and nature of changes in GI activity following the receipt of the COVID-19 vaccine was assessed in the form of the following side effects: more frequent bowel movements, looser stool, harder stool, mix of diarrhea and constipation, more gas, presence of mucus in stool, presence of blood in stool, and more pain while passing stool. Both the onset of and the duration of the side effects was reported in the following increments, respectively: 1–2 days, 2–5 days, 1–2 weeks, more than a month, or N/A if not experienced; 1–2 days, 3–7 days, 1–2 weeks, 2–4 weeks, 1–3 months, more than 3 months, or N/A if not experienced. The frequency of stomach pain and presence of nausea following receipt of the COVID-19 vaccine was investigated through respondent reporting of presence, onset, and duration of these changes.

The final section of the questionnaire considered changes in GI activity during and/or following contraction of COVID-19 infection. Respondents were first asked to report the date of their most recent contraction and then were inquired about both the presence and duration of the following GI side effects: more frequent bowel movements, looser stool, harder stool, mix of diarrhea and constipation, more gas, presence of mucus in stool, presence of blood in stool, more pain while passing stool.

## Ethics Statement

The study protocol (TSH OOR 202201, WIRB Work Order #1-1557933-1) was reviewed and approved as exempt by the Stamford Hospital's Institutional Review Board of record. Individual participants were required to consent to participate prior to completing the survey.

## CRediT authorship contribution statement

**Kaly D. Houston:** Data curation, Investigation, Methodology, Resources, Writing – review & editing. **Josette Hartnett:** Data curation, Investigation, Methodology, Project administration, Resources, Supervision, Writing – review & editing. **Suzanne J. Rose:** Data curation, Investigation, Methodology, Project administration, Resources, Supervision, Writing – review & editing.

## Declaration of Competing Interest

The authors declare that they have no known competing financial interests or personal relationships which have or could be perceived to have influenced the work reported in this article. This research did not receive any specific grant from funding agencies in the public, commercial, or not-for-profit sectors.

## Data Availability

Investigating the Prevalence of Gastrointestinal Symptomology Following Receipt of the COVID-19 Vaccine (Original data) (ICPSR).

## Appendix Supplementary materials

Image, application 1

Image, application 2
