# Supplementary material for: Investigating the association between the COVID-19 vaccination and incident gastrointestinal symptomology: A comprehensive dataset
Source: Data Brief. 2023 Jun 1;48:109287. doi: 10.1016/j.dib.2023.109287 (PMC10232932; doi:10.1016/j.dib.2023.109287)
Supplement: Supplementary file 2 [file mmc2.docx]

**PAGE 1: WELCOME/INTRODUCTION**

[informed consent]

**PAGE 2: VACCINATION STATUS**

1. Have you received at least one COVID-19 vaccination?
   1. Yes
   2. No 🡪 SKIP TO END OF SURVEY

**PAGE 3: DEMOGRAPHICS**

1. What is your age?
   1. 0 - 15 years old
   2. 16 - 30 years old
   3. 31 - 45 years old
   4. 46-65 years old
   5. 65+ years old
   6. Prefer not to answer
2. What is your BMI (body mass index)? If unknown, click here to calculate your BMI: <https://www.nhlbi.nih.gov/health/educational/lose_wt/BMI/bmicalc.htm>
   1. Less than 18.5 (underweight)
   2. 18.5-24.9 (ideal weight)
   3. 25-29.9 (overweight)
   4. 30-34.9 (moderate obesity)
   5. 35-40 (obesity)
   6. More than 40 (morbidly obese)
3. What gender do you identify as?
   1. Male
   2. Female
   3. Prefer not to say
   4. Other (please specify)
4. Are you married?
   1. Yes
   2. No
   3. Prefer not to say
5. Please specify your ethnicity.
   1. Caucasian
   2. African-American
   3. Hispanic or Latino
   4. Asian
   5. Native American
   6. Native Hawaiian or Pacific Islander
   7. Two or More
   8. Other/Unknown
   9. Prefer not to say
6. What is the highest degree or level of education you have completed?
   1. Some High School
   2. High School
   3. Bachelor's Degree
   4. Master's Degree
   5. Ph.D. or higher

F. Trade School

G. Prefer not to say

1. Have you ever been diagnosed with Inflammatory Bowel Disease and/or Irritable Bowel Syndrome (IBS)?
   1. No 🡪 SKIP TO PAGE 6
   2. IBS only
   3. IBD only (ulcerative colitis)
   4. IBD only (Crohn’s disease)
   5. Both IBS and IBD (ulcerative colitis)
   6. Both IBS and IBD (Crohn’s disease)

**PAGE 4: MEDICAL DIAGNOSIS**

1. In what year were you diagnosed with IBS? (If you have not been diagnosed with IBS, leave field blank)

(YYYY)

- 1. ___________

1. In what year were you diagnosed with IBD? (If you have not been diagnosed with IBS, leave field blank)

(YYYY)

- 1. ___________

**PAGE 5:**

**Page description:** A “flare” is defined as the reappearance or worsening of existing disease symptoms.

1. Did you experience a “flare” of your IBD or IBS symptoms following any doses of the COVID-19 vaccine? (If you did not receive a given dose, please select “N/A”)

|  | Yes | No | N/A |
| --- | --- | --- | --- |
| Dose 1 |  |  |  |
| Dose 2 |  |  |  |
| Dose 3 (Booster) |  |  |  |
| Dose 4 (Booster) |  |  |  |

1. Did you experience a “flare” of your IBD or IBS symptoms following contraction of a COVID-19 infection? (If you did not contract COVID-19, please select “N/A”)
   1. Yes
   2. No
   3. N/A

**PAGE 6: COVID-19 VACCINATION INFORMATION**

1. Vaccination 1: What was the date of your first dose? (MM/YYYY)
   1. ______________
2. Vaccination 1: Who was the manufacturer?
   1. Pfizer-BioNTech
   2. Moderna
   3. Johnson & Johnson
   4. Other (please specify)
3. Vaccination 2:
4. I did not receive a second dose 🡪 SKIP TO PAGE 10
5. What was the date of your second dose? (MM/YYYY)
   1. ______________

**PAGE 7: COVID-19 VACCINATION INFORMATION**

1. Vaccination 2: Who was the manufacturer?
   1. Pfizer-BioNTech
   2. Moderna
   3. Johnson & Johnson
   4. Other (please specify)
2. Vaccination 3 (Booster):
3. I did not receive a third dose 🡪 SKIP TO PAGE 10
4. What was the date of your third dose? (MM/YYYY)
   1. ______________

**PAGE 8: COVID-19 VACCINATION INFORMATION**

1. Vaccination 3: Who was the manufacturer?
   1. Pfizer-BioNTech
   2. Moderna
   3. Johnson & Johnson
   4. Other (please specify)
2. Vaccination 4 (Booster):
3. I did not receive a fourth dose 🡪 SKIP TO PAGE 10
4. What was the date of your fourth dose? (MM/YYYY)
   1. ______________

**PAGE 9: COVID-19 VACCINATION INFORMATION**

1. Vaccination 4: What was the manufacturer of the dose?
   1. Pfizer-BioNTech
   2. Moderna
   3. Johnson & Johnson
   4. Other (please specify)

**PAGE 10: SYMPTOMOLOGY POST-COVID-19 VACCCINATION**

1. Did you experience any side effects following any doses of the COVID-19 vaccine?
   1. Yes
   2. No 🡪 SKIP TO PAGE 17

**PAGE 11: SYMPTOMOLOGY POST-COVID-19 VACCCINATION**

1. Indicate the dose(s) that resulted in you experiencing the following common side effects: (If you did not experience a given side effect, please select "N/A" on the dropdown menu) *dropdown separates out by dose*
   1. Reaction at the injection arm (e.g. pain, redness, swelling)
   2. Tiredness throughout your body
   3. Headache
   4. Muscle pain throughout your body
   5. Chills
   6. Fever

Drop down menu options:

- 1. N/A (I did not experience this side effect)
  2. Dose 1 only
  3. Dose 2 only
  4. Dose 3 only
  5. Dose 4 only
  6. Dose 1 & 2
  7. Dose 1 & 3
  8. Dose 1 & 4
  9. Dose 2 & 3
  10. Dose 2 & 4
  11. Dose 3 & 4
  12. Dose 1, 2 & 3
  13. Dose 1, 2 & 4
  14. Dose 1, 3, & 4
  15. Dose 2, 3 & 4
  16. Dose 1-4

1. On average, how long did these side effects last? (If you did not experience any side effects following a given dose or did not receive the dose, please select "N/A" in the dropdown menu) *dropdown separates out by dose*

|  | Duration of side effects |
| --- | --- |
| Dose 1 | *Dropdown menu |
| Dose 2 | “” “” |
| Dose 3 (Booster) | “” “” |
| Dose 4 (Booster) | “” “” |

Drop down menu options:

- 1. N/A
  2. 1 or 2 days following vaccination
  3. 3-7 days following vaccination
  4. More than a week following vaccination

1. Do you recall experiencing a change in gastrointestinal activity following any doses of the COVID-19 vaccine?
   1. Yes
   2. No 🡪 SKIP TO PAGE 13

**PAGE 12: SYMPTOMOLOGY POST-COVID-19 VACCCINATION: GASTROINTESTINAL ACTIVITY**

1. Indicate which of the following doses resulted in changes in gastrointestinal activity. (If you did not receive a given dose, please select “N/A”)

|  | Yes | No | N/A |
| --- | --- | --- | --- |
| Dose 1 |  |  |  |
| Dose 2 |  |  |  |
| Dose 3 (Booster) |  |  |  |
| Dose 4 (Booster) |  |  |  |

1. Indicate the dose(s) that resulted in you experiencing the following common side effects: (If you did not experience a given side effect, please select "N/A" on the dropdown menu) *dropdown separates out by dose*
   1. More frequent bowel movements
   2. Looser stool (or more diarrhea)
   3. Harder stool (or more constipation)
   4. Mix of diarrhea and constipation
   5. More gas
   6. Presence of mucus in stool
   7. Presence of blood in stool
   8. More pain while passing stool

Drop down menu options:

1. N/A (I did not experience this side effect)
2. Dose 1 only
3. Dose 2 only
4. Dose 3 only
5. Dose 4 only
6. Dose 1 & 2
7. Dose 1 & 3
8. Dose 1 & 4
9. Dose 2 & 3
10. Dose 2 & 4
11. Dose 3 & 4
12. Dose 1, 2 & 3
13. Dose 1, 2 & 4
14. Dose 1, 3, & 4
15. Dose 2, 3 & 4
16. Dose 1-4
17. When did these changes in gastrointestinal activity start occur, following vaccination? (If you did not experience any GI changes following a given dose or did not receive the dose, please select "N/A" in the dropdown menu)

|  | Onset of changes in GI activity |
| --- | --- |
| Dose 1 | *Dropdown menu |
| Dose 2 | “” “” |
| Dose 3 (Booster) | “” “” |
| Dose 4 (Booster) | “” “” |

Drop down menu options:

- 1. N/A
  2. 1-2 days following vaccination
  3. 2-5 days following vaccination
  4. 1-2 weeks following vaccination
  5. More than a month following vaccination

1. How long did these changes in gastrointestinal activity last? (If you did not experience any GI changes following a given dose or did not receive the dose, please select "N/A" in the dropdown menu)

|  | Duration of changes in GI activity |
| --- | --- |
| Dose 1 | *Dropdown menu |
| Dose 2 | “” “” |
| Dose 3 (Booster) | “” “” |
| Dose 4 (Booster) | “” “” |

Drop down menu options:

- 1. N/A
  2. 1-2 days
  3. 3-7 days
  4. 1-2 weeks
  5. 2-4 weeks
  6. 1-3 months
  7. More than 3 months

**PAGE 13: SYMPTOMOLOGY POST-COVID-19 VACCCINATION: STOMACH PAIN**

1. Do you recall experiencing new stomach pain or a change in frequency of stomach pain following any doses of the COVID-19 vaccine?
   1. Yes
   2. No 🡪 SKIP TO PAGE 15

**PAGE 14: SYMPTOMOLOGY POST-COVID-19 VACCCINATION: STOMACH PAIN**

1. Indicate which of the following doses resulted in changes in frequency of stomach pain. (If you did not receive a given dose, please select “N/A”)

|  | Yes | No | N/A |
| --- | --- | --- | --- |
| Dose 1 |  |  |  |
| Dose 2 |  |  |  |
| Dose 3 (Booster) |  |  |  |
| Dose 4 (Booster) |  |  |  |

1. When did this change in frequency of stomach pain start to occur, following vaccination? (If you did not experience any changes following a given dose or did not receive the dose, please select "N/A" in the dropdown menu)

|  | Onset of changes in frequency of stomach pain |
| --- | --- |
| Dose 1 | *Dropdown menu |
| Dose 2 | “” “” |
| Dose 3 (Booster) | “” “” |
| Dose 4 (Booster) | “” “” |

Drop down menu options:

- 1. N/A
  2. 1-2 days following vaccination
  3. 3-7 days following vaccination
  4. 1-2 weeks following vaccination
  5. 2-4 weeks following vaccination
  6. More than a month following vaccination

1. How long did these changes in frequency of stomach pain last? (If you did not experience any changes following a given dose or did not receive the dose, please select "N/A" in the dropdown menu)

|  | Duration of changes in frequency of stomach pain |
| --- | --- |
| Dose 1 | *Dropdown menu |
| Dose 2 | “” “” |
| Dose 3 (Booster) | “” “” |
| Dose 4 (Booster) | “” “” |

Drop down menu options:

- 1. N/A
  2. 1-2 days
  3. 3-7 days
  4. 1-2 weeks
  5. 2-4 weeks
  6. 1-3 months
  7. More than 3 months

**PAGE 15: SYMPTOMOLOGY POST-COVID-19 VACCCINATION: NAUSEA**

1. Do you recall experiencing a change in frequency of nausea following any doses of the COVID-19 vaccine?
   1. Yes
   2. No 🡪 SKIP TO PAGE 17

**PAGE 16: SYMPTOMOLOGY POST-COVID-19 VACCCINATION: NAUSEA**

1. Indicate which of the following doses resulted in changes in frequency of nausea. (If you did not receive a given dose, please select “N/A”)

|  | Yes | No | N/A |
| --- | --- | --- | --- |
| Dose 1 |  |  |  |
| Dose 2 |  |  |  |
| Dose 3 (Booster) |  |  |  |
| Dose 4 (Booster) |  |  |  |

1. When did this change in frequency of nausea start to occur, following vaccination? (If you did not experience any changes following a given dose or did not receive the dose, please select "N/A" in the dropdown menu)

|  | Onset of changes in frequency of nausea |
| --- | --- |
| Dose 1 | *Dropdown menu |
| Dose 2 | “” “” |
| Dose 3 (Booster) | “” “” |
| Dose 4 (Booster) | “” “” |

Drop down menu options:

- 1. N/A
  2. 1-2 days following vaccination
  3. 3-7 days following vaccination
  4. 1-2 weeks following vaccination
  5. 2-4 weeks following vaccination
  6. More than a month following vaccination

1. How long did these changes in frequency of nausea last? (If you did not experience any changes following a given dose or did not receive the dose, please select "N/A" in the dropdown menu)

|  | Duration of changes in frequency of nausea |
| --- | --- |
| Dose 1 | *Dropdown menu |
| Dose 2 | “” “” |
| Dose 3 (Booster) | “” “” |
| Dose 4 (Booster) | “” “” |

Drop down menu options:

1. N/A
2. 1-2 days
3. 3-7 days
4. 1-2 weeks
5. 2-4 weeks
6. 1-3 months
7. More than 3 months

**PAGE 17: COVID-19 INFECTION SYMPTOMOLOGY**

1. Have you contracted COVID-19?
   1. No 🡪 SKIP TO END OF SURVEY
   2. Yes (please specify the date of your most recent contraction, MM/YYYY)
   3. _______________

**PAGE 18: COVID-19 INFECTION SYMPTOMOLOGY: GASTROINTESTINAL ACTIVITY**

1. Do you recall experiencing a change in bowel movement habits during or following COVID-19 infection?
   1. No 🡪 SKIP TO END OF SURVEY
   2. Yes, during acute infection
   3. Yes, after acute infection
   4. Yes, during and after acute infection

**PAGE 19: COVID-19 INFECTION SYMPTOMOLOGY: GASTROINTESTINAL ACTIVITY**

1. What was the nature of the change in gastrointestinal activity following contraction of COVID-19?
   1. More frequent bowel movements
   2. Looser stool (or more diarrhea)
   3. Harder stool (or more constipation)
   4. Mix of diarrhea and constipation
   5. More gas
   6. Presence of mucus in stool
   7. Presence of blood in stool
   8. More pain while passing stool
2. How long did these changes in gastrointestinal activity last?
   1. 1 or 2 days
   2. 2-7 days
   3. 1-2 weeks
   4. 2-4 weeks
   5. 1-3 months
   6. More than 3 months

**PAGE 20:**

1. Please enter any additional comments you may have below:
   1. ___________________________________________

**END OF SURVEY**

Thank you for taking the time to complete this survey. We truly value the information you have provided. Your responses will contribute to our research investigating the impact of COVID-19 vaccination on gastrointestinal symptomology.

If you have any questions or comments or wish to contact the study’s research staff, please email Dr. Suzanne Rose, Executive Director of Research at Stamford Health, SRose@stamhealth.org
